# Impact of Oral Pre-Exposure Secretory IgA Prophylactic Produced in Rice on Gut Microbiome Homeostasis

**DOI:** 10.3390/pharmaceutics18040457

**Published:** 2026-04-09

**Authors:** Ravi Bharadwaj, Carlos Gaspar, Tyler D. Moeller, Doyle Ward, Mark S. Klempner, Yang Wang, Lisa A. Cavacini

**Affiliations:** 1 Division of Infectious Diseases and Immunology, Department of Medicine, University of Massachusetts Chan Medical School, Worcester, MA 01605, USA; 2 Vysnova Partners LLC, Alexandria, VA 22314, USA; 3 U.S. Naval Medical Research Unit SOUTH, Lima 07031, Peru; 4 Department of Microbiology and Physiological Systems, University of Massachusetts Chan Medical School, Worcester, MA 01605, USA; 5 Division of Innate Immunity, Department of Medicine, University of Massachusetts Chan Medical School, Worcester, MA 01605, USA

**Keywords:** secretory IgA, gut microbiome, oral immunotherapy

## Abstract

**Background/Objectives**: Enterotoxigenic *Escherichia coli* (ETEC) is a leading cause of diarrheal illness worldwide, resulting in approximately 380,000 deaths annually, with significant morbidity in children and travelers to endemic regions. ETEC infection begins with the attachment of the bacterium to the small intestine via filamentous colonization factors (CF), followed by the production of heat-labile (LT) and heat-stable (ST) toxins that induce watery diarrhea. Targeting CF to prevent ETEC attachment is challenging due to strain heterogeneity. **Methods**: In previous studies, we developed a class-switched human monoclonal antibody, 68–90, expressed as secretory IgA (SIgA) in rice for cost-effective and stable storage. Rice-produced SIgA exhibited comparable binding efficiency to CfaE, a component of CF, compared to CHO-produced SIgA in vitro. **Results**: In this work, we showed that oral administration of 68–90 SIgA to *Aotus nancymaae* did not alter gut microbiome distribution or show signs of systemic exposure. **Conclusions**: These findings suggest that oral delivery of ETEC-specific SIgA is safe and does not disrupt the gut microbial population, highlighting its potential as an effective and targeted therapeutic strategy.

## 1. Introduction

Enterotoxigenic *Escherichia coli* (ETEC) is one of the most common causes of diarrheal illness in the developing world. Based on WHO reports, ETEC-related diarrhea is one of the leading causes of death in children under five years old in developing countries. Even if a child survives ETEC infection, there are long-lasting effects throughout life, including malnutrition and impaired cognitive development [1,2,3,4,5]. An estimated ten million cases per year occur among travelers and military personnel deployed in endemic regions [4]. In fact, infectious diarrhea historically has been a substantial cause of morbidity for deployed U.S. warfighters, with ETEC as one of the most prevalent pathogens, and continues to affect those currently serving overseas in the global war on terror. Traditionally, infected adults have been given short courses of antibiotics to which the ETEC strain is sensitive. This can shorten the duration and volume of diarrhea. However, ETEC strains are becoming increasingly resistant to antibiotics [6,7,8].

ETEC is a non-invasive pathogen that mediates small intestine adherence through filamentous bacterial surface structures known as colonization factors (CF). Once bound to the small intestine, the bacteria produce heat-labile toxins (LT) and heat-stable toxins (ST) that, through a cascade process, cause a net flow of water from the cell into the intestinal lumen, resulting in watery diarrhea [7,9]. The attachment and colonization steps are critical for bacteria to effectively produce toxins and represent a potential strategic target for preventing ETEC infection. Development of a vaccine against bacterial attachment and colonization factors has long been considered an effective approach against ETEC diarrhea. One of the main challenges to developing vaccines against colonization factors is the heterogeneity of ETEC strains [5,10,11,12]. Theoretically, a vaccine against all fimbrial adhesins will be effective as a pre-exposure immunoprophylaxis for diarrheal diseases caused by major pathogenic ETEC strains. However, it is unclear whether a recombinant protein containing domains of all adhesion subunits can be stably engineered without a prohibitive cost for manufacturing. Moreover, the protein domain of the adhesin responsible for the protective immune response is not clear. As such, alternative approaches are warranted.

Oral administration of hyperimmune bovine colostrum, generated by immunization of cows with a mixture of ETEC antigens, prevented or ameliorated signs and symptoms resulting from ETEC challenge of healthy subjects [13,14]. Previously, our laboratory identified a large panel of human monoclonal antibodies against CfaE, the adhesin subunit of fimbriae CFA/I of strain H10407, the most commonly used ETEC strain in laboratory and clinical studies [15]. To improve gastrointestinal tract stability and function, we have shown that subcutaneous (subQ) administration of dimeric IgA or oral administration of secretory IgA to CfaE prevents intestinal colonization in mice and non-human primates challenged with H10407 [16]. One of these human monoclonal antibodies (68–90) has been produced in rice (manuscript in preparation) as a secretory IgA (SIgA). It is expected that 68–90 SIgA will prevent ETEC infection in nonhuman primates and humans, following challenge.

In assessing an effective prophylactic or therapeutic measure, it is important to consider the gut microbiota. The gut microbiota is a diverse community of microorganisms, including bacteria, archaea, and eukarya, residing within the gastrointestinal (GI) tract [17,18,19,20,21]. Various factors such as diet, ethnicity, age, lifestyle changes, and medication use can influence the composition of the gut microbiota [19]. Dysbiosis, or an imbalance in the gut microbial population, can contribute to various diseases affecting the immune system, nervous system, and cardiovascular health [17,20,21].

Oral administration is the most common and practical route for drug delivery due to its safety, convenience, cost-effectiveness, flexibility, and high patient compliance [22]. Drugs administered orally often encounter the gut microbiome, especially those absorbed in the lower GI tract, potentially affecting the microbial composition. While it is well-established that antibiotics can impact gut microbes, recent research reveals that over 24% of non-antibiotic drugs exhibit antibiotic-like effects on the gut microbiota [23]. In this context, pathogen-specific monoclonal antibodies have emerged as a promising alternative to broad-spectrum antibiotics. These antibodies demonstrate the potential to combat specific pathogens without disrupting the overall fecal microbiome. Monoclonal antibodies [24] are an attractive choice due to their precision in targeting, extended half-life, and ability to synergize with the host’s immune response [25]. While the global burden of ETEC is well documented and pathogen-specific monoclonal antibodies offer targeted alternatives to broad-spectrum antibiotics, it remains unknown whether oral, antigen-specific 68-90 SIgA can inhibit ETEC without perturbing the commensal gut microbiome. Building on our prior functional studies demonstrating 68–90 SIgA efficacy against ETEC [16,26], we hypothesized that oral 68–90 SIgA would preserve gut microbial diversity and community structure while restricting ETEC. To test this, we conducted a longitudinal pre/post analysis of fecal microbiota during 68–90 SIgA treatment using 16S rRNA sequencing and paired beta diversity.

## 2. Materials and Methods

### 2.1. Expression and Purification of 68–90 SIgA

Human monoclonal antibody, 68–90, has been described previously [15,16]. As a comparator for rice-expressed antibody for in vitro experiments, 68–90 was expressed as a human IgA antibody in CHO cells, as previously described [16]. Others have used rice to produce VHH antibodies, and it represents a low-cost, alternative production platform [27,28]. Here, plasmids used for CHO-expressed SIgA were codon optimized for plants and were used to transform rice cultivars. (manuscript in preparation). Work to characterize the product for plant-specific N-glycans (e.g., β1,2-xylose, core α1,3-fucose) is ongoing. Since the product is administered orally, it can be argued that this is not different than if rice itself were consumed. 68–90 SIgA, from either CHO harvests or rice extracts, was purified by an immunoaffinity column for use in this study. As assessed by SEC-HPLC, 89% was SIgA, with the remaining protein monomeric IgA. Immunoreactivity of purified antibody was confirmed by ELISA reactivity with CfaE (see below).

### 2.2. CfaE ELISA

On the prior day, 2 μg/mL CfaE was coated on high-binding 96-well plates overnight at 4 °C. The next day, plates were washed three times with PBS with 0.05% tween 20 and blocked with BSA in PBS for 2 h at room temperature. Serial dilutions of purified antibody, fecal extracts, or serum samples were added to the blocked plates after washing. After incubation for 1 h at room temperature, plates were washed, and goat anti-human IgA was added at 1:30,000 dilution in PBS for 1 h at room temperature. Plates were washed, and 100 μL/well TMB solution was added for 2 min, and the reaction was stopped with 85% phosphoric acid. Plates were read at 450 nm for absorption, and 600 nm for wavelength correction, and relative optical density was computed in Microsoft Excel.

### 2.3. Processing and Testing of Fecal Samples

To check the presence of 68–90 SIgA, 100 mg fecal pellets were resuspended and processed individually in IgA extraction buffer (0.05% PBST, 0.05% fetal calf serum, 1 mg/mL EDTA and protease cocktail). After 15 min incubation on ice, a homogenous suspension was prepared by vertexing. Then it was centrifuged at 23,000 *g* for 15 min at 4 °C. Supernatant devoid of fecal debris was collected and assayed for CfaE ELISA as mentioned above.

### 2.4. Library Preparation and Sequencing

For microbiome analysis, DNA was extracted using a ZymoBIOMICS DNA miniprep kit (D4300, Zymo Research, Irvine, CA, USA), and metagenomic DNA sequencing libraries were constructed using the Nextera XT DNA Library Prep Kit (FC-131-1096, Illumina, San Diego, CA, USA) and sequenced on a NextSeq2000 Sequencing System as 2 × 150 nucleotide paired-end reads.

### 2.5. Sequence Analysis

Shotgun metagenomic reads were first trimmed and quality filtered to remove sequencing adapters and host contamination using Trimmomatic [29] and Bowtie2 [30], respectively, as part of the KneadData (version = 0.12.2) pipeline [31] using NCBI RefSeq assembly GCF_000952055.2 (*A. nancymaae*) as host reference. Metagenomic data was profiled for microbial taxonomic abundances Metaphlan (version = 4.1.1) [31] with database mpa_vOct22_CHOCOPhlAnSGB_202212.

### 2.6. Statistical Analysis

Analyses were performed in QIIME 2 (https://qiime2.org, accessed on 3 March 2026) and R (https://www.R-project.org/, accessed on 14 December 2025) [32]. Beta diversity was evaluated using Bray–Curtis and Jaccard (composition-based) and weighted and unweighted UniFrac (phylogeny-informed) distance matrices, followed by principal coordinates analysis (PCoA) for visualization. Within-animal compositional change was quantified as the pre/post-pairwise distance for each metric. Global shifts between pre and post were tested with PERMANOVA (vegan::adonis2, 9999 permutations) with permutations stratified by animal ID to respect pairing; pseudo-F, R^2^, and adjusted *p*-values are reported. Assumptions were checked by testing homogeneity of multivariate dispersions (vegan::betadisper/PERMDISP); when dispersion differed, results were interpreted cautiously and complemented by comparing within-animal distances using paired tests. Where applicable, distance-based redundancy analysis (db-RDA) was used to assess the contribution of covariates (e.g., batch/cage) with permutations constrained by animal ID.

Taxonomic summaries (phylum to genus) were generated from the ASV table. Exploratory paired pre/post changes in relative abundance were assessed with nonparametric paired tests and FDR correction; to address compositionality, we complemented these with a compositional method (e.g., ANCOM-BC) including animal ID as a blocking factor. Effect sizes are presented as median paired differences and clr-scale changes where applicable.

Baseline variability and dispersion were assessed using distance-to-centroid analyses (PERMDISP; vegan::betadisper) for PRE and POST periods, with significance evaluated by permutation tests (permutest). Within-animal variability was quantified as the distribution of day-to-day pairwise distances during the PRE period and compared to PRE–POST paired distances for each animal using paired tests (normality by Shapiro–Wilk; paired *t*-test or Wilcoxon signed-rank). Multiple comparisons were controlled using Benjamini–Hochberg FDR. Effect sizes (Hedges’ g or matched-pairs rank-biserial correlation) and 95% CIs are reported.

Sensitivity analyses were conducted across rarefaction depths and on non-rarefied, transformed data (e.g., centered log-ratio) to confirm robustness of beta diversity results. All plots (PCoA, within-animal distance “spaghetti” plots) were generated in R (phyloseq, vegan, ggplot2). No formal power calculation was performed. The design comprised five pre-treatment and five post-treatment sampling days per animal (*n* = 5). Global differences were tested via repeated-measures PERMANOVA (adonis2) with permutations constrained by animal ID (pre vs. post as the primary factor). Multiple comparisons were controlled with Benjamini–Hochberg FDR, and we report exact R^2^, *p*, and q values. Day effects nested within period were explored descriptively; inference on treatment focuses on the animal-level paired design.

### 2.7. Animal Use and Welfare

The animal study was conducted in an AAALAC-accredited facility in compliance with the Animal Welfare Act and other applicable U.S. Government statutes. All methods were carried out in accordance with relevant guidelines and regulations and in accordance with ARRIVE guidelines. This study was approved by the U.S. Naval Medical Research Unit SOUTH (NAMRU SOUTH) IACUC (Protocol N6 22-04), U.S. Navy Bureau of Medicine and Surgery Veterinary Affairs Office, and Peruvian Servicio National Forestal y de Fauna Silvestre.

The *A. nancymaae* used in this study were purchased from the Instituto Veterinario de Investigaciones Tropicales y de Altura (IVITA), University Nacional Mayor de San Marcos, Lima, Peru, and transported to NAMRU SOUTH in Lima, Peru. Animals were randomly selected from a cohort of animals transferred to the facility for several studies. The animals were identified by unique tattoo numbers on their abdomens and were maintained in pairs except when required to be individually housed for study sample collection. Animals had a mean (±standard deviation) weight of 886 ± 68 g and a mean age of 38 ± 16 months. Animals were fed a standard non-human primate diet supplemented with fruit and provided water ad libitum. Following the study, animals were returned to IVITA.

### 2.8. Administration of 68–90 SIgA

**Figure 1:**
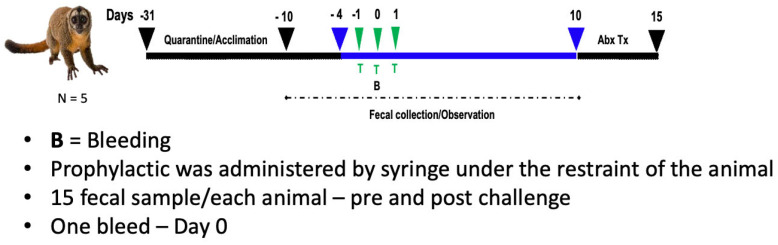
Study design for oral treatment of 68–90 SIgA in *A. nancymaae*. T = 10 mg/kg oral dose of 68–90 SIgA on indicated days. D-01, D0, D01 are days when oral treatment of 68–90 SIgA was administrated. B = Blood collected on indicated day. The fecal matter collection time interval is indicated by black dotted arrows line. The black line indicates the quarantine/acclimation period, pair-house. The blue line indicates pre- and post-treatment observation, single house. The study start and end are indicated by black arrows. Timepoints are labeled PRE1–PRE5 (pre-treatment) and POST1–POST5 (post-treatment); D0 indicates the dosing day. Legacy labels (e.g., PRE01/P01/A01) have been harmonized to this scheme.

A dose of 10 mg/kg of SIgA antibody was selected as this has been shown to be protective in prior studies using nonhuman primates [16]. SIgA antibody (10 mg/Kg) was resuspended in 5 mL phosphate-buffered saline (PBS) supplemented with 5% sucrose to promote palatability. The diluted antibody was administered orally on days 1–3 with a syringe by manually restraining the animal. All animals were observed for 10 days after SIgA administration, according to the ETEC challenge diarrhea model in *A. nancymaae* previously described [16]. For blood collection (day 1; after 24 h of first dose of 68–90 SIgA), the animals were anesthetized with ketamine hydrochloride (10 mg/kg weight, Ketalar, Parke-Davis). Blood samples were collected and processed further individually. For the analysis of the microbiota, fecal samples were collected during the quarantine period (day-15 and -11), during the observation period pre-administration of SIgA (day-8 and -5), during and after the SIgA administration (day 1, 2, 3, 5, 8 and 10). The treatment study design is illustrated in Figure 1. Since all animals received SIgA, blinding was not possible. No formal a priori or post hoc power calculation was performed. Given the paired design with five animals (five pre and five post-timepoints per animal) and FDR control across metrics, the study primarily has sensitivity to detect large compositional shifts. Results are interpreted using exact R^2^, *p*-values, and FDR-adjusted q-values.

## 3. Results

### 3.1. Measures of Non-Human Primate Serum and Feces Following 68–90 SIgA Oral Administration

In this study, five *A. nancymaae* non-human primates (NHP) received oral gavage of 10 mg/kg 68–90 SIgA on days 1–3 (D-01, D0, D01) (Figure 1). Serum samples were collected on day 2 (D0) to assess the presence of 68–90 SIgA. The lower limit of assay detection was 130 pg/mL. 68–90 SIgA was not detected in any of the serum samples (Table 1). In the absence of 68–90 SIgA in *A. nancymaae* sera, we could not conduct further analyses, including calculating pharmacokinetic parameters like AUC and oral bioavailability. To ensure consistency, we use PRE1–PRE5 for the five pre-treatment fecal collection days and POST1–POST5 for the five post-treatment days. The dosing day is denoted D-0 (administration of 68–90 SIgA); it is not used as a fecal timepoint label. Legacy labels used during data processing map to the final scheme as follows: Day −01 ≡ PRE1; Day +01 ≡ POST1; PRE01–PRE05 ≡ PRE1–PRE5; P01–P05 ≡ PRE1–PRE5; A01–A05 ≡ POST1–POST5.

Fecal samples were collected at the same time points as serum to determine if 68–90 SIgA was in the samples. Interestingly, the presence of 68–90 SIgA was detected in the fecal samples of all animals across these three days but was cleared by day 6 (D05) (Table 1). This finding suggests that 68–90 SIgA remains localized within the gastrointestinal tract and does not breach the intestinal epithelial barrier. These results indicate minimal safety concerns with oral administration of 68–90 SIgA, supporting its potential as a promising candidate for targeted therapies.

### 3.2. Effect of Oral 68–90 SIgA on the Microbiota

**Figure 2:**
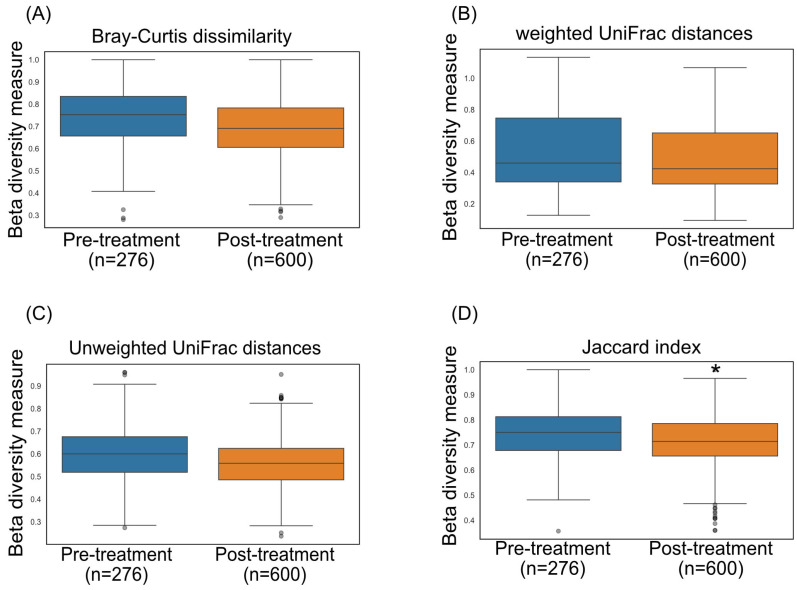
Microbiome analysis of Aotus monkey fecal samples following oral treatment with VENBETA6890 IgA. Beta diversity was assessed using the PERMANOVA method and calculated using four different metrics: (**A**) Bray-Curtis dissimilarities, (**B**) Weighted UniFrac distances, (**C**) Unweighted UniFrac distances, and (**D**) Jaccard index. R^2^ and q-values are summarized in Table 2. * *p* < 0.05. Details of comparison tests are described in method section.

While oral antibiotics are known to influence the gut microbiome, research indicates that antibody-based drugs demonstrate safety and do not disrupt the microbial population [25]. To validate the impact of 68–90 SIgA on the gut microbiome, a 10 mg/kg dosage was administered on days 1–3(D-01, D0, D01) (Figure 1). Fecal samples were collected from five *A. nancymaae* at 11 timepoints between 15 days before and 10 days after the oral administration of 68–90 SIgA (Figure 1). Shotgun metagenomic sequencing was used to profile fecal microbial communities, and beta diversity was assessed using Bray–Curtis dissimilarity, weighted and unweighted UniFrac distances, and the Jaccard index. (Figure 2) Beta diversity was assessed by PERMANOVA (999 permutations; permutations constrained by animal ID) across four metrics. To control for multiple comparisons across metrics and time points, *p*-values were adjusted using the Benjamini–Hochberg false discovery rate (FDR); exact R^2^ and FDR-adjusted q-values are reported in Table 2 (and summarized in the figure legends).

Given the known sensitivity of presence/absence metrics to rare taxa, we analyzed Jaccard and unweighted UniFrac on rarefied, filtered data (20,000 reads; ≥10% (prevalence; ≥0.05% abundance). Where binary and abundance-weighted metrics differed, or signals were borderline, effect sizes were small (R^2^ < 1%) and/or not FDR-significant; we therefore prioritized interpretations supported by abundance-weighted metrics and tempered conclusions accordingly.

**Figure 3:**
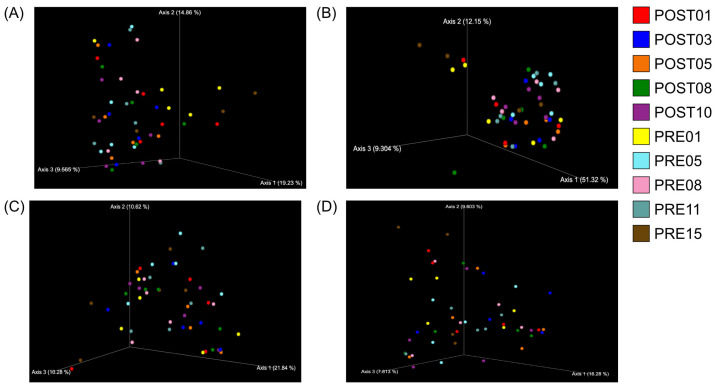
Principal Coordinates Analysis (PCoA) of beta diversity for PRE vs POST using (**A**) Bray–Curtis, (**B**) weighted UniFrac, (**C**) unweighted UniFrac, and (**D**) Jaccard. Each point is a daily sample (five PRE and five POST per animal); points from the same animal are connected to indicate pre→post displacement. Shaded ellipses (95% confidence areas) summarize within-period dispersion. Visual inspection shows comparable PRE and POST spread and small within-animal displacement relative to baseline scatter. Exact PERMANOVA effect sizes (R^2^) and FDR-adjusted q-values are reported in Table 2.

**Figure 4:**
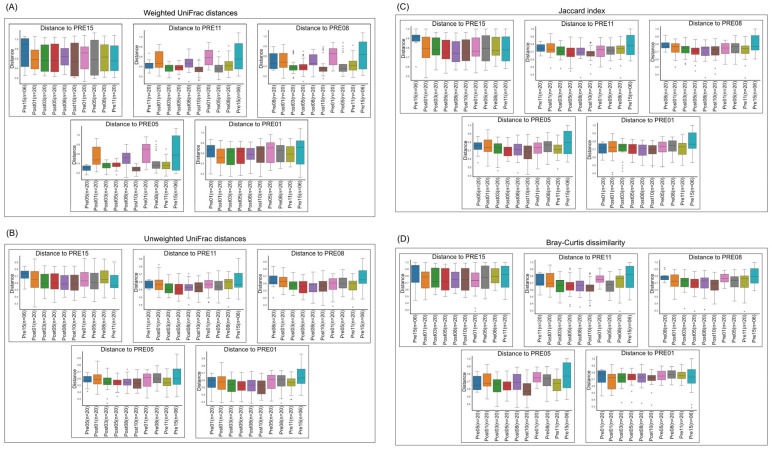
Beta diversity analysis comparing microbiome composition before and after oral treatment using the PERMANOVA method and pseudo-F test. Diversity metrics include: (**A**) Weighted UniFrac distances, (**B**) Unweighted UniFrac distances, (**C**) Jaccard index, and (**D**) Bray-Curtis dissimilarities. Each comparison was made between a specific day prior to treatment and corresponding post-treatment time points. R^2^ and q-values are summarized in the Supplementary Tables. Details of comparison tests are described in method section.

Across metrics, effect sizes were very small (Bray–Curtis R^2^ = 0.0029 [0.29% variance explained], *p* = 0.053; weighted UniFrac R^2^ = 0.0028 [0.28%], *p* = 0.051; unweighted UniFrac R^2^ = 0.0058 [0.58%], *p* = 0.229), indicating negligible separation between pre- and post-treatment communities. The Jaccard index showed a nominal *p* = 0.013 with a correspondingly small R^2^ (see Table 2 for the exact R^2^ and q-value). After FDR correction, results were interpreted based on the q-values reported in Table 2. Across metrics, PCoA showed PRE and POST samples intermingled with comparable within-period spread; within-animal pre→post displacement vectors were small relative to the baseline [32] day-to-day scatter, consistent with very small global effect sizes (Figure 3A–D) (R^2^ < 1%; Table 2). Consistent with these small effect sizes, daily within-animal shifts did not exceed baseline day-to-day variability and were not statistically significant after FDR adjustment (Supplementary Tables S1–S4). Paired PERMANOVA comparisons of specific pre- vs. post-treatment days similarly showed no evidence of large compositional shifts; where *p* or q values were borderline, the variance explained remained <1%, and conclusions have been tempered accordingly (Figure 4A–D).

Comprehensive pairwise pre/post comparisons at the ASV/taxon level and across specific days (Supplementary Tables S1–S4) showed that the vast majority of contrasts were not significant after Benjamini–Hochberg FDR correction (q < 0.05). Isolated nominal *p* < 0.05 findings were either not FDR significant or associated with very small effects and lacked consistency across animals and timepoints. These pairwise results align with the global beta diversity findings (PERMANOVA R^2^ uniformly < 1%) and support the interpretation that no large, systematic compositional shifts were detected following 68–90 SIgA treatment.

**Figure 5:**
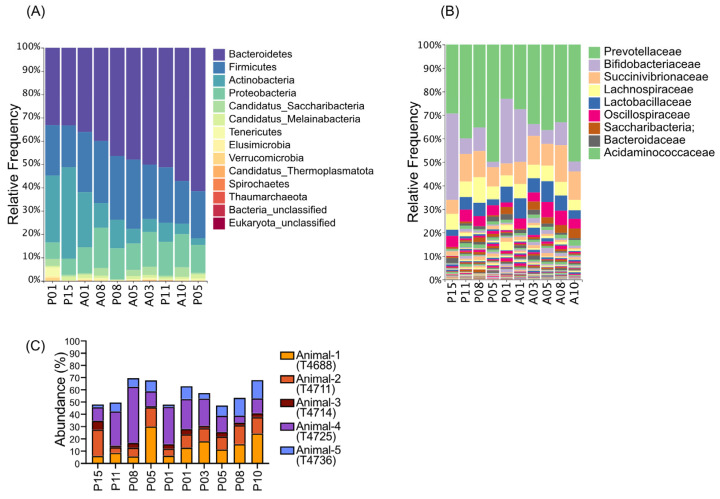
Taxonomic profiles of *A. nancymaae* fecal samples before and after oral treatment with VB6890 IgA. (**A**) Relative abundances of major bacterial phyla, (**B**) Relative abundances at the family level, and (**C**) Changes within Proteobacterial species.

In the taxonomy profile, the fecal microbiota of naïve animals before treatment primarily consisted of bacteria from the phyla Bacteroidetes, Firmicutes, Actinobacteria, and Proteobacteria (Figure 5A). At the family level, Prevotellaceae, Bifidobacteriaceae, and Succinivibrionaceae were predominant (Figure 5B). A similar taxonomic composition was observed after 68–90 SIgA treatment, except on days P01 and A01, which showed an increased presence of the Bifidobacteriaceae family. However, this returned to the previous taxonomic distribution on day A05. Proteobacteria are typically found in low abundance within the healthy gut microbiota; however, changes in their levels are often considered a marker of gut dysbiosis. Such microbial imbalances have been linked to various health conditions, including inflammation and metabolic disturbances [33]. Since many members of the Proteobacteria family share structural features with surface proteins of ETEC, there is a possibility of cross-reactivity with 68–90 SIgA. Nevertheless, our detailed analysis of Proteobacterial taxa revealed no significant differences between samples collected before and after antibody treatment (Figure 5C). These findings suggest that oral treatment with 68–90 SIgA does not have a significant impact on the gut microbiome population.

## 4. Discussion

Conventional thinking is that antibodies, or any other orally delivered therapeutic biological molecules, will be degraded or destroyed in the digestive tract. However, nature has clearly demonstrated that antibodies, if of the appropriate molecular structure, can be delivered effectively to the gut in colostrum or breast milk to protect against neonatal infections. While there are other immune components to breast milk such as cytokines, antibodies represent a major component with secretory antibody being the predominant functional immunoglobulin. The multiple glycans contributed by the secretory component, the polymerization by the J chain, as well as the glycans on the IgA molecules themselves, all contribute to the functional activity by protecting immunoglobulin from degradation, trapping targets within the mucus, and protecting the epithelial cell barrier.

Orally administered recombinant SIgA has been shown to protect against ETEC in a nonhuman primate model [16,34]. SIgA-based camelid antibody fusions can protect piglets against ETEC when delivered orally in feed [35,36]. In humans, oral administration of hyperimmune bovine colostrum prevented or ameliorated signs and symptoms resulting from ETEC (H10407) challenge of healthy subjects [13,14,37,38]. A freeze-dried anti-rotavirus nanobody (ARP-1) given orally has been shown to reduce stool output in a multi-dose trial in Bangladesh [39]. Despite these developments for oral biologics, little is known of the effect on the microbiome.

While antibiotics remain a cornerstone of bacterial infection treatment, their broad-spectrum activity often leads to unintended disruption of the gut microbiota and can contribute to the emergence of antibiotic-resistant organisms. In contrast, mAb offers a more targeted therapeutic strategy, binding specifically to pathogen-associated antigens and minimizing off-target effects. This specificity enables mAb to facilitate pathogen clearance without significantly impacting commensal microbial communities.

In our study, we evaluated the effects of oral administration of 68–90 SIgA—a monoclonal antibody targeting ETEC—on the gut microbiome of *A. nancymaae*. Even at a relatively high dose of 10 mg/kg, 68–90 SIgA showed minimal perturbation of gut microbial composition. To comprehensively assess microbiome changes pre- and post-treatment, we applied four widely used beta diversity metrics: Bray–Curtis dissimilarity, Weighted UniFrac, Unweighted UniFrac, and Jaccard index. Nonetheless, interpretation is constrained by the absence of a concurrent placebo (PBS) control group; with a small cohort and a within-animal pre/post design, natural temporal fluctuations in the Aotus microbiome cannot be fully excluded, and causal inference is limited. Future randomized, placebo-controlled studies with denser longitudinal sampling will be needed to confirm these findings.

Bray–Curtis dissimilarity, which assesses differences in the relative abundance of shared microbial species, revealed low dissimilarity scores, indicating high similarity between microbial communities before and after treatment. Likewise, the Weighted UniFrac metric—which incorporates both phylogenetic information and relative abundance—did not detect any significant shifts in microbial composition, supporting the conclusion that 68–90 SIgA administration does not alter the dominant bacterial taxa.

The Unweighted UniFrac metric, which considers only the presence or absence of microbial taxa regardless of abundance, also revealed no significant differences between pre- and post-treatment samples. We acknowledge a statistically significant Jaccard signal after FDR correction (q = 0.013). Given that Jaccard is a presence/absence metric sensitive to low-abundance detections, and that abundance-weighted metrics (Bray–Curtis, weighted UniFrac) were not FDR-significant, we interpret this as a statistically detectable but biologically minor shift. A plausible explanation is a transient change in rare taxa coincident with SIgA delivery; limited cross-reactivity with commensal Proteobacteria may contribute. Consistent with this, MaAsLin2 identified a small number of species-level increases post-treatment (e.g., Parasutterella excrementihominis), suggesting subtle, feature-specific adjustments rather than broad community restructuring. Larger, randomized studies with denser longitudinal sampling will be needed to determine the reproducibility and mechanism of these subtle signals. Notably, metrics that emphasize microbial abundance (Bray–Curtis and Weighted UniFrac) consistently found no significant differences, strengthening the conclusion that 68–90 SIgA spares the overall microbiota structure. Although five pre and five post-timepoints per animal improved precision and allowed characterization of day-to-day variability, the effective unit for treatment inference is the animal, and small effects may remain undetected. Observed effect sizes were <1%, supporting the absence of large, FDR-significant shifts, while acknowledging that subtle changes cannot be excluded.

To further validate these findings, we compared microbial profiles from individual pre-treatment days to each of the post-treatment days (Figure 3). This day-by-day comparison using all four beta diversity metrics, including the Jaccard index, reinforced the absence of significant changes, confirming the robustness of the microbiome preservation observed with 68–90 SIgA treatment. The concordance between minimal global effect sizes and predominantly non-significant pairwise comparisons (Supplementary Tables S1–S4) reinforces that any treatment-related microbiome changes, if present, are small and not robust to multiplicity correction. Together, these results support a key advantage of pathogen-specific antibody therapies over traditional antibiotics: the ability to effectively target infectious agents while preserving gut microbiota integrity. This is particularly relevant for diseases where microbiome health is critical to recovery and long-term immunity.

In addition to the microbiome outcomes, we note that GI stability of 68–90 SIgA in simulated gastric/intestinal conditions and under protease challenge, as well as structural integrity and aggregation control by orthogonal analytics, were established in our prior work [16,37]. In the current study, we used a PBS + 5% sucrose formulation based on its stabilizer/osmolyte properties and practicality for oral dosing. Serum analyses at 24 h showed no detectable SIgA, suggesting any systemic exposure is low and/or transient. Together, these data support a luminal, antigen-specific mechanism with a microbiome-sparing profile. Translationally, protective oral delivery strategies (e.g., enteric coatings, microencapsulation, localized pH buffering, or mucoadhesive systems) merit evaluation to enhance gastric transit and small intestinal residence, and longer or repeated dose studies with denser PK/PD sampling will be important to confirm durability, efficacy, and microbiota sparing benefits over time. Although the treatment duration used for 68–90 SIgA was similar to standard antibiotic regimens, the antibody demonstrated rapid systemic clearance, potentially reducing the risk of long-term side effects often associated with prolonged exposure to biologics. Future studies assessing longer or repeated mAb administration schedules will be important to further evaluate durability, efficacy, and microbiota-sparing benefits over time.

Similarly, other mAbs targeting major human pathogens like *Staphylococcus aureus, Pseudomonas aeruginosa, Clostridioides difficile*, and *Klebsiella pneumoniae* exhibited minimal effects on the gut microbiome compared to conventional antibiotics [25,40,41,42]. Moreover, systemic biologics used in IBD (e.g., anti-TNF, anti-IL-12/23, vedolizumab) have been associated with microbiome shifts largely secondary to modulation of mucosal inflammation and barrier function [43,44,45]. In contrast, our oral, antigen-specific SIgA acts luminally to restrict ETEC and, consistent with this mechanism, we observed minimal pre/post changes in community structure, supporting a microbiome-sparing profile. The gut microbial population acts as a physical barrier, impeding pathogen colonization through space limitation and antimicrobial compound production. The symbiotic relationship between the immune system and gut microbiota shapes a healthy gut microbial ecosystem, whereas dysbiosis is associated with various metabolic and inflammatory diseases. Despite dense longitudinal sampling (five pre- and five post-treatment days per animal), inference on the treatment effect is anchored at the animal level (*n* = 5). No a priori power calculation was performed. We therefore emphasize effect sizes and FDR-adjusted q-values in interpreting results. Observed PERMANOVA effect sizes were uniformly small (R^2^ < 1%), supporting the absence of large, community-wide shifts; however, the study is underpowered to detect small effects, and subtle changes cannot be excluded. Our findings demonstrate that the oral administration of pathogen-specific mAb, 68–90 SIgA, does not perturb the gut microbial population in a non-human primate model, underscoring its potential as an effective and targeted therapeutic approach.

## Figures and Tables

**Table 1:** Detection of 68–90 SIgA in fecal and serum samples of *A. nancymaae* following oral administration. 68–90 SIgA was detectable in fecal samples from Day-1 through Day 3 post-treatment, indicating gastrointestinal presence. 68–90 SIgA was not detected in serum at any time point, suggesting lack of systemic absorption. (ND = not detected).

Samples	D-11	68–90 SIgA Treatment				D 05
		D-01	D-0		D +01	
	Feces	Feces	Feces	Serum	Feces	Feces
Animal-1 (4688)	ND	0.031 mg/mL	0.33 mg/mL	ND	0.024 mg/mL	ND
Animal-2 (4711)	ND	0.021 mg/mL	0.35 mg/mL	ND	0.021 mg/mL	ND
Animal-3 (4714)	ND	0.019 mg/mL	0.3 mg/mL	ND	0.035 mg/mL	ND
Animal-4 (4725)	ND	0.015 mg/mL	0.32 mg/mL	ND	0.029 mg/mL	ND
Animal-5 (4736)	ND	0.027 mg/mL	0.77 mg/mL	ND	0.027 mg/mL	ND

**Table 2:** Beta diversity analysis of fecal microbiome before and after treatment of 68–90 SIgA. Bray–Curtis Dissimilarity: Measures the compositional dissimilarity between different groups based on abundance data. Weighted UniFrac Distances: A metric that considers both the phylogenetic relationships and the relative abundance of taxa. Unweighted UniFrac Distances: Evaluate differences between microbial communities using only the presence or absence of taxa while considering their phylogenetic relationships. Jaccard Index: Measures community similarity and diversity based on the presence or absence of taxa, focusing solely on species composition without considering abundance or phylogenetic information. Analyses were conducted on 49 samples due to exclusion of one sample that failed the pre-specified QC threshold (20,000 reads). All PERMANOVA tests used 999 permutations constrained by animal ID.

Analysis	Group 1	Group 2	Sample Size	Permutations	Pseudo-F	*p*-Value	q-Value
Bray–Curtis dissimilarity	POST	PRE	49	999	1.8393	0.049	0.051
weighted UniFrac distances	POST	PRE	49	999	1.2806	0.229	0.229
unweighted UniFrac distances	POST	PRE	49	999	1.7716	0.043	0.052
Jaccard index	POST	PRE	49	999	1.9051	0.013	0.013

## Data Availability

The datasets generated and analyzed during the current study are available in the BioProject database, ID PRJNA1260583. The reviewer link (not public until published) is: https://dataview.ncbi.nlm.nih.gov/object/PRJNA1260583?reviewer=c6jv0jctl54o858gsl7vcg956p, accessed on 30 November 2025.

## Supplementary Materials

The following supporting information can be downloaded at: https://www.mdpi.com/article/10.3390/pharmaceutics18040457/s1, Table S1: Weighted UniFrac distances. Table S2: Unweighted UniFrac distances. Table S3: Jaccard index. Table S4: Bray-Curtis dissimilarity.

## Abbreviations

The following abbreviations are used in this manuscript:

ETEC	Enterotoxigenic *Escherichia coli*
CF	colonization factors
LT	heat-labile toxin
ST	heat-stable toxin
SIgA	secretory IgA
subQ	subcutaneous
GI	gastrointestinal
mAb	monoclonal antibody
IVITA	Instituto Veterinario de Investigaciones Tropicales y de Altura
