# Supplementary material for: Impact of Oral Pre-Exposure Secretory IgA Prophylactic Produced in Rice on Gut Microbiome Homeostasis
Source: Pharmaceutics. 2026 Apr 9;18(4):457. doi: 10.3390/pharmaceutics18040457 (PMC13119238; doi:10.3390/pharmaceutics18040457)
Supplement: Supplementary file 1 [file pharmaceutics-18-00457-s001.zip › pharmaceutics-4173696-supplementary.pdf]

Supplementary Table S1: Weighted UniFrac distances

Group 1	Group 2	Sample size	Permutations	pseudo-F	p-value	q-value
POST01	POST03	10	999	0.5407	0.9	0.985
	POST05	10	999	0.6559	0.96	0.985
	POST08	10	999	0.7957	0.775	0.985
	POST10	10	999	0.8876	0.612	0.985
	PRE01	10	999	0.5705	0.904	0.985
	PRE05	10	999	0.7100	0.894	0.985
	PRE08	10	999	0.5428	0.97	0.985
	PRE11	10	999	0.6460	0.939	0.985
	PRE15	9	999	0.6511	0.873	0.985
POST03	POST05	10	999	0.2972	0.954	0.985
	POST08	10	999	0.6998	0.779	0.985
	POST10	10	999	0.8664	0.552	0.985
	PRE01	10	999	1.2381	0.226	0.985
	PRE05	10	999	1.0014	0.442	0.985
	PRE08	10	999	1.0537	0.408	0.985
	PRE11	10	999	1.0116	0.491	0.985
	PRE15	9	999	1.2838	0.145	0.985
POST05	POST08	10	999	0.2662	0.949	0.985
	POST10	10	999	0.6238	0.78	0.985
	PRE01	10	999	1.3383	0.067	0.985
	PRE05	10	999	1.0645	0.312	0.985
	PRE08	10	999	0.7254	0.877	0.985
	PRE11	10	999	0.8973	0.62	0.985
	PRE15	9	999	1.1140	0.351	0.985
POST08	POST10	10	999	0.4720	0.88	0.985
	PRE01	10	999	1.1647	0.227	0.985
	PRE05	10	999	1.0342	0.44	0.985
	PRE08	10	999	0.9162	0.542	0.985
	PRE11	10	999	1.0205	0.433	0.985
	PRE15	9	999	1.0740	0.342	0.985
POST10	PRE01	10	999	1.1804	0.266	0.985
	PRE05	10	999	0.8228	0.685	0.985
	PRE08	10	999	0.9972	0.392	0.985
	PRE11	10	999	0.8777	0.664	0.985
	PRE15	9	999	1.1610	0.243	0.985
PRE01	PRE05	10	999	0.8784	0.621	0.985
	PRE08	10	999	0.8689	0.669	0.985
	PRE11	10	999	0.8727	0.625	0.985
	PRE15	9	999	1.0091	0.495	0.985
PRE05	PRE08	10	999	0.7618	0.834	0.985
	PRE11	10	999	0.6168	0.985	0.985
	PRE15	9	999	0.9632	0.508	0.985
PRE08	PRE11	10	999	0.5163	0.938	0.985
	PRE15	9	999	0.8478	0.769	0.985
PRE11	PRE15	9	999	0.8102	0.818	0.985

Supplementary Table S2: Unweighted UniFrac distances

Group 1	Group 2	Sample size	Permutations	pseudo-F	p-value	q-value
POST01	POST03	10	999	0.4904	0.865	0.99
	POST05	10	999	0.6976	0.875	0.99
	POST08	10	999	0.6749	0.889	0.99
	POST10	10	999	0.7072	0.777	0.99
	PRE01	10	999	0.3117	0.961	0.99
	PRE05	10	999	0.7637	0.738	0.99
	PRE08	10	999	0.5122	0.928	0.99
	PRE11	10	999	0.5019	0.985	0.99
	PRE15	9	999	0.6743	0.822	0.99
POST03	POST05	10	999	0.2995	0.974	0.99
	POST08	10	999	0.3970	0.939	0.99
	POST10	10	999	0.4294	0.944	0.99
	PRE01	10	999	0.9644	0.475	0.890625
	PRE05	10	999	1.0805	0.409	0.890625
	PRE08	10	999	1.1869	0.261	0.890625
	PRE11	10	999	0.8253	0.602	0.99
	PRE15	9	999	1.1696	0.265	0.890625
POST05	POST08	10	999	0.0926	0.99	0.99
	POST10	10	999	0.4209	0.898	0.99
	PRE01	10	999	1.0940	0.297	0.890625
	PRE05	10	999	1.5141	0.097	0.890625
	PRE08	10	999	0.9785	0.456	0.890625
	PRE11	10	999	1.0949	0.418	0.890625
	PRE15	9	999	1.2964	0.146	0.890625
POST08	POST10	10	999	0.2805	0.921	0.99
	PRE01	10	999	0.9505	0.443	0.890625
	PRE05	10	999	1.2793	0.213	0.890625
	PRE08	10	999	1.0110	0.434	0.890625
	PRE11	10	999	1.0421	0.437	0.890625
	PRE15	9	999	1.1382	0.267	0.890625
POST10	PRE01	10	999	0.9196	0.467	0.890625
	PRE05	10	999	1.0338	0.413	0.890625
	PRE08	10	999	1.1050	0.307	0.890625
	PRE11	10	999	1.0323	0.453	0.890625
	PRE15	9	999	1.1028	0.32	0.890625
PRE01	PRE05	10	999	1.3034	0.179	0.890625
	PRE08	10	999	0.7157	0.744	0.99
	PRE11	10	999	0.9182	0.55	0.95192308
	PRE15	9	999	1.2490	0.236	0.890625
PRE05	PRE08	10	999	1.1152	0.312	0.890625
	PRE11	10	999	0.6792	0.77	0.99
	PRE15	9	999	1.1116	0.29	0.890625
PRE08	PRE11	10	999	0.6600	0.779	0.99
	PRE15	9	999	1.0608	0.41	0.890625
PRE11	PRE15	9	999	0.9149	0.537	0.95192308

Supplementary Table S3: Jaccard index

Group 1	Group 2	Sample size	Permutations	pseudo-F	p-value	q-value
POST01	POST03	10	999	0.5407	0.9	0.985
	POST05	10	999	0.6559	0.96	0.985
	POST08	10	999	0.7957	0.775	0.985
	POST10	10	999	0.8876	0.612	0.985
	PRE01	10	999	0.5705	0.904	0.985
	PRE05	10	999	0.7100	0.894	0.985
	PRE08	10	999	0.5428	0.97	0.985
	PRE11	10	999	0.6460	0.939	0.985
	PRE15	9	999	0.6511	0.873	0.985
POST03	POST05	10	999	0.2972	0.954	0.985
	POST08	10	999	0.6998	0.779	0.985
	POST10	10	999	0.8664	0.552	0.985
	PRE01	10	999	1.2381	0.226	0.985
	PRE05	10	999	1.0014	0.442	0.985
	PRE08	10	999	1.0537	0.408	0.985
	PRE11	10	999	1.0116	0.491	0.985
	PRE15	9	999	1.2838	0.145	0.985
POST05	POST08	10	999	0.2662	0.949	0.985
	POST10	10	999	0.6238	0.78	0.985
	PRE01	10	999	1.3383	0.067	0.985
	PRE05	10	999	1.0645	0.312	0.985
	PRE08	10	999	0.7254	0.877	0.985
	PRE11	10	999	0.8973	0.62	0.985
	PRE15	9	999	1.1140	0.351	0.985
POST08	POST10	10	999	0.4720	0.88	0.985
	PRE01	10	999	1.1647	0.227	0.985
	PRE05	10	999	1.0342	0.44	0.985
	PRE08	10	999	0.9162	0.542	0.985
	PRE11	10	999	1.0205	0.433	0.985
	PRE15	9	999	1.0740	0.342	0.985
POST10	PRE01	10	999	1.1804	0.266	0.985
	PRE05	10	999	0.8228	0.685	0.985
	PRE08	10	999	0.9972	0.392	0.985
	PRE11	10	999	0.8777	0.664	0.985
	PRE15	9	999	1.1610	0.243	0.985
PRE01	PRE05	10	999	0.8784	0.621	0.985
	PRE08	10	999	0.8689	0.669	0.985
	PRE11	10	999	0.8727	0.625	0.985
	PRE15	9	999	1.0091	0.495	0.985
PRE05	PRE08	10	999	0.7618	0.834	0.985
	PRE11	10	999	0.6168	0.985	0.985
	PRE15	9	999	0.9632	0.508	0.985
PRE08	PRE11	10	999	0.5163	0.938	0.985
	PRE15	9	999	0.8478	0.769	0.985
PRE11	PRE15	9	999	0.8102	0.818	0.985

Supplementary Table S4: Bray-Curtis dissimilarity

Group 1	Group 2	Sample size	Permutations	pseudo-F	p-value	q-value
POST01	POST03	10	999	0.9636	0.509	0.954375
	POST05	10	999	0.6377	0.892	0.959
	POST08	10	999	0.6215	0.846	0.959
	POST10	10	999	1.4712	0.077	0.954375
	PRE01	10	999	0.5413	0.867	0.959
	PRE05	10	999	1.1237	0.371	0.954375
	PRE08	10	999	0.6753	0.861	0.959
	PRE11	10	999	1.1023	0.363	0.954375
	PRE15	9	999	0.7745	0.563	0.959
POST03	POST05	10	999	0.1981	0.959	0.959
	POST08	10	999	0.5241	0.899	0.959
	POST10	10	999	0.9469	0.476	0.954375
	PRE01	10	999	1.5994	0.115	0.954375
	PRE05	10	999	0.9999	0.446	0.954375
	PRE08	10	999	0.9848	0.442	0.954375
	PRE11	10	999	0.7810	0.719	0.959
	PRE15	9	999	1.6192	0.198	0.954375
POST05	POST08	10	999	0.2563	0.957	0.959
	POST10	10	999	0.7494	0.658	0.959
	PRE01	10	999	1.5605	0.13	0.954375
	PRE05	10	999	0.8518	0.629	0.959
	PRE08	10	999	0.5601	0.91	0.959
	PRE11	10	999	0.8167	0.661	0.959
	PRE15	9	999	1.3875	0.308	0.954375
POST08	POST10	10	999	0.8333	0.631	0.959
	PRE01	10	999	1.0923	0.341	0.954375
	PRE05	10	999	1.0879	0.388	0.954375
	PRE08	10	999	0.5781	0.956	0.959
	PRE11	10	999	0.6488	0.922	0.959
	PRE15	9	999	0.9438	0.431	0.954375
POST10	PRE01	10	999	2.2165	0.034	0.954375
	PRE05	10	999	0.9468	0.504	0.954375
	PRE08	10	999	1.0928	0.361	0.954375
	PRE11	10	999	0.8565	0.666	0.959
	PRE15	9	999	1.9974	0.138	0.954375
PRE01	PRE05	10	999	1.6224	0.128	0.954375
	PRE08	10	999	1.0390	0.353	0.954375
	PRE11	10	999	1.3563	0.194	0.954375
	PRE15	9	999	0.5065	0.877	0.959
PRE05	PRE08	10	999	0.7264	0.746	0.959
	PRE11	10	999	0.6229	0.822	0.959
	PRE15	9	999	1.3870	0.364	0.954375
PRE08	PRE11	10	999	0.4336	0.89	0.959
	PRE15	9	999	0.9394	0.463	0.954375
PRE11	PRE15	9	999	1.0950	0.397	0.954375
